# Untangling discrepancies between cerebrovascular autoregulation correlation coefficients: An exploration of filters, coherence and power

**DOI:** 10.14814/phy2.70332

**Published:** 2025-04-17

**Authors:** Stefan Yu Bögli, Ihsane Olakorede, Erta Beqiri, Giada Cucciolini, Virginia Motroni, Claudia Ann Smith, Marina Sandra Cherchi, Ronan O'Leary, Peter Smielewski

**Affiliations:** ^1^ Brain Physics Laboratory, Division of Neurosurgery, Department of Clinical Neurosciences University of Cambridge Cambridge UK; ^2^ Department of Neurology and Neurocritical Care Unit, Clinical Neuroscience Center University Hospital Zurich, University of Zurich Zurich Switzerland; ^3^ Department of Surgical, Medical, Molecular Pathology and Critical Care Medicine University of Pisa Pisa Italy; ^4^ Departmental Structure of Neuroanesthesia and Critical Care Azienda Ospedaliero‐Universitaria Pisana Pisa Italy; ^5^ Department of Critical Care Marques de Valdecilla University Hospital, and Biomedical Research Institute (IDIVAL) Santander Cantabria Spain; ^6^ Neurosciences and Trauma Critical Care Unit Addenbrooke's Hospital, Cambridge University Hospitals Cambridge UK

**Keywords:** cerebrovascular autoregulation, intensive care, multimodality monitoring, traumatic brain injury

## Abstract

Cerebrovascular autoregulation (CAR) maintains stable cerebral perfusion by adjusting arteriole diameters in response to slow pressure fluctuations. Various CAR correlation coefficients—PRx (based on intracranial pressure—ICP), Mx (based on transcranial doppler—TCD), and COx/THx (based on near‐infrared spectroscopy—NIRS)—are used interchangeably despite fundamental differences. 566 hours of ICP, NIRS, and TCD data from 38 traumatic brain injury (TBI) patients were assessed. The intercorrelation between CAR correlation coefficients was compared in relation to: (1) The impact of different filtering methods (to minimize noise); (2) The impact of slow wave power (i.e., magnitude of incoming trigger); (3) The impact of coherence (i.e., to what extent can the power of slow waves explain the change in power within the cerebral biosignal). Only coherence stratification consistently increased the metric intercorrelation to PRx (high vs. low) when evaluating Mx (0.43 vs. 0.08, *p* < 0.01) and THx (0.36 vs. 0.05, *p* < 0.01). Additionally, high coherence and ABP power were associated with fewer correlation coefficients around 0. Coherence increases the intercorrelation between the different CAR metrics. These sections might be regarded as more reliable, since they are derived from different biosignals that are all affected by CAR through different mechanisms.

## 1 INTRODUCTION

Cerebrovascular autoregulation (CAR) refers to the brain's ability to maintain stable cerebral blood flow (CBF) and consequently adequate oxygen and nutrient supply despite changes in cerebral perfusion (CPP) pressure (Lassen, 1964). Failure can lead to hypo‐ or hyper‐perfusion exacerbating or causing brain injury (Al‐Kawaz et al., 2022; Czosnyka & Miller, 2014). In intensive care management of acute brain injuries such as TBI (traumatic brain injury) its value for treatment personalization is underscored by consensus statements (Depreitere et al., 2021; Le Roux et al., 2014; Park et al., 2023). Several modalities are used to quantify the state of CAR (Zeiler, Donnelly, Calviello, et al., 2017) including intracranial pressure (ICP) monitoring, transcranial doppler (TCD) ultrasonography, or near‐infrared spectroscopy (NIRS). For estimation, various methods can be employed (Czosnyka et al., 2009; Klein et al., 2019) from the most simple widely applied methods such as mean arterial pressure challenge (Hawryluk et al., 2019; Kunapaisal et al., 2024) to more complex measures derived from transfer function analysis (Panerai et al., 2023) (which allows for the assessment of gain and phase shift to characterize the change in cerebral blood flow velocity (CBFV) in response to changes in arterial blood pressure (ABP)), and lastly the continuous CAR correlation coefficients (Zeiler, Donnelly, Calviello, et al., 2017) which are explored in the current manuscript and represent the most commonly used proxy measures for dynamic assessment within intensive care. All CAR correlation coefficients explored in this manuscript are described in detail in Table 1 and assess the relationship between the source of variability (slow changes in ABP or CPP) and a CBF proxy measure. Of note, there is no definitive consensus whether one method of CAR monitoring is universally superior to the others (Depreitere et al., 2021). Each metric has its strengths and limitations, and the choice often depends on the clinical context and the resources available. While the Pressure Reactivity Index (PRx—derived from ICP) is the most widely applied continuous proxy measure of CAR (Czosnyka et al., 1997), it requires placement of an invasive ICP probe, which is reserved for more severely affected patients. Mx (Czosnyka et al., 1996), on the other hand, requires TCD assessments which need to be performed by skilled personnel (Bögli et al., 2024). NIRS might be most widely applicable (allowing for the assessment of COx and THx—derived from rSO_2_ and hemoglobin content, respectively), due to its noninvasive and easy‐to‐apply properties, but suffers from patient‐specific physiology dependent issues (Owen‐Reece et al., 1999).

**TABLE 1:** Explored cerebrovascular autoregulation proxy measures.

Coefficient^b^	Signals^a^	Modality	Strengths	Limitations
PRx (Czosnyka et al., 1997; Zeiler, Donnelly, Menon, et al., 2017)	ABP and ICP (intracerebral pressure)	ICP transducer	Most researched continuous index in TBI; continuous; global measure	Necessitates invasive surgical placement of ICP transducer; represents a surrogate of cerebral blood volume (i.e., cerebrovascular reactivity) rather than CAR
Mx/Mxa (Czosnyka et al., 1996; Zeiler, Donnelly, Menon, et al., 2017)	ABP or CPP and CBFV (cerebral blood flow velocity)	TCD	Direct representation of CAR	Operator‐dependent necessitating skilled personnel; intermittent; reflects focal CAR (vascular territory of the middle cerebral artery)
COx/COxa (Brady et al., 2007; Zeiler, Donnelly, Menon, et al., 2017)	ABP or CPP and rSO_2_ (regional oxygen saturation)	NIRS	Non‐invasive and easy‐to‐apply; continuous	Limited by physiological factors incl. extracranial contamination, skin color, limited depth of penetration and differences in arterio‐venous blood measured; reflects focal CAR (frontal cortex); rSO_2_ is seen as a proxy measure of CBF, but is also affected by ICP
THx/THxa (Highton et al., 2015; Zeiler, Donnelly, Menon, et al., 2017)	ABP or CPP and CHB (total hemoglobin)	NIRS	See COx/COxa	See COx/COxa; CHB is seen as a proxy measure of cerebral blood volume
DOHx/DOHxa (Highton et al., 2012)	ABP or CPP and HHB (deoxygenated hemoglobin)	NIRS	See COx/COxa	See COx/COxa; HHB is seen as a measure of arteriovenous blood transfer; limited research in adult TBI
OHx/OHxa (Sainbhi et al., 2023)	ABP or CPP and O_2_HB (oxygenated hemoglobin)	NIRS	See COx/COxa	See COx/COxa; O_2_Hb is seen as a measure of arteriovenous blood transfer; limited research in adult TBI
DHx/DHxa (Sainbhi et al., 2023)	ABP or CPP and DHB (difference oxygenated deoxygenated hemoglobin)	NIRS	See COx/COxa	See COx/COxa; DHB is seen as a measure of arteriovenous blood transfer; limited research in adult TBI

Abbreviations: ABP, arterial blood pressure; CAR, cerebrovascular autoregulation; CBF, cerebral blood flow; CBFV, cerebral blood flow velocity; CHB, total hemoglobin; COx, cerebral oximetry index; CPP, cerebral perfusion pressure; DC, decompressive craniectomy; DHB, difference oxygenated deoxygenated hemoglobin; DHx, delta hemoglobin index; DOHx, deoxygenated hemoglobin index; GOS, Glasgow outcome scale; HHB, deoxygenated hemoglobin; ICU, intensive care unit; ICP, intracranial pressure; Mx, mean flow index; NIRS, near infrared spectroscopy; O_2_HB, oxygenated hemoglobin; OHx, oxygenated hemoglobin reactivity index; PRx, pressure reactivity index; rSO_2_, regional cerebral oxygen saturation; TBI, traumatic brain injury; TCD, transcranial doppler sonography; THx, total hemoglobin index.

^a^ All the different indices are calculated by estimating the pearson correlation coefficient considering 10 s average values of ABP or CPP and either cerebral measures within a 5‐min data window updated every minute.

^b^ Coefficients followed by a lowercase a represent the indices calculated with ABP (e.g., Mxa) as compared to CPP (e.g., Mx).

The current analysis relies on the assumption that all the measured changes in biosignals within the frequency range explored represent downstream effects of CAR. The CAR mechanism itself causes a change in arteriolar diameter, a measure which can only be explored invasively (Klein et al., 2022). All the biosignals explored (e.g., ICP, CBFV, and rSO_2_) represent secondary downstream changes caused by the resulting change in resistance. Building on the hypothesis that periods during which distinct CAR correlation coefficients exhibit higher intercorrelation—reflecting a consistent CAR state irrespective of the biosignal explored—may yield more reliable assessments, our study sought to determine whether the intercorrelation among measures derived from different biosignals could be enhanced by applying specific filtering techniques or by slow wave power or coherence based stratification.

## 2 MATERIALS AND METHODS

This is a retrospective data analysis on records stored in the Brain Physics database (REC 23/YH/0085) which was approved by the local ethics committee (Yorkshire & The Humber—Leeds East Research Ethics Committee). Multimodality monitoring was performed as part of the local clinical practice. TCD was performed as part of a prospective clinical audit (Clinical Project ID4201—TCD based assessment of intracranial hemodynamics in patients admitted to NCCU). Informed consent was waived by the local authorities.

### 2.1 Patient inclusion and data acquisition

Patients inclusion and data acquisition was previously described (Bögli et al., 2024). In short, all TBI patients (*n* = 74) who required ICP‐directed therapy were assessed for inclusion. If deemed suitable (i.e., there are no clear exclusion criteria met for the application of TCD such as severe skull fractures, wounds, or lesions within the area of insonation, insufficient bone window for high quality data acquisition), patients underwent extended daily TCD monitoring. Thirty‐eight patients were included in the final sample. The following high‐resolution waveform signals (100 Hz or higher sampling rate) were acquired and analyzed: ABP, ICP, bilateral CBFV, bilateral rSO_2_, bilateral changes in oxygenated/deoxygenated/total hemoglobin concentrations (O_2_HB, HHB, and CHB, respectively). The devices used were NIRS—Root Monitoring Platform using O3 Regional Oximetry (Masimo, CA, United States); TCD—Delica EMS 9D System (Shenzen Delica Medical Equipment Co. Ltd., China) or Doppler Box (DWL Compumedics, Singen, Germany); intraparenchymal ICP transducer—Codman ICP MicroSensor (Codman & Shurtleff, Massachusetts, US); ABP line—Codman ICP MicroSensor® (Codman&Shurtleff, Massachusetts, US). The signals from the different devices were integrated using the ICM+ software (Cambridge Enterprise Ltd., Cambridge, UK).

### 2.2 Clinical descriptors

Age, sex, mechanism of injury, type of primary brain injury, differentiation isolated TBI versus polytrauma, post‐resuscitation Glasgow Coma Score, incidence of decompressive craniectomy (DC), and 6‐month outcome (assessed using the Glasgow Outcome Scale—GOS) were retrieved from the database.

### 2.3 Data preprocessing

Data preprocessing and artifact removal were performed as previously described (Bögli et al., 2024). Briefly, the following artifacts were removed for the different signals: ABP—Sections with arterial line failure or manipulation; ABP below 0 mmHg or above 300 mmHg and/or with a pulse amplitude below 15 mmHg. ICP—ICP below −30 mmHg or above 200 mmHg and/or with low amplitude (<0.04 mmHg) and/or with a 95% Spectral edge frequency above 10 Hz (representing pure noise). CBFV—Sections with low signal‐to‐noise ratio or missing pulsatile flow signal.

### 2.4 Calculation of correlation coefficients

The different correlation coefficients explored are described in Table 1. All of these were calculated based on standardized methodology as previously described (Zeiler, Donnelly, Calviello, et al., 2017; Zeiler, Donnelly, Menon, et al., 2017). Each correlation coefficient reflects the Pearson correlation coefficient computed from 30 consecutive 10‐s averages of two signals: an input (either ABP or CPP) and a brain biosignal output (i.e., ICP, CBFV, rSO_2_, CHB, HHB, O_2_HB, and DHB). To capture dynamic temporal trends, this calculation is repeated every 60 s, resulting in coefficients with an 80% overlap. The indices explored were as follows: pressure reactivity index PRx, mean flow index Mx, cerebral oximetry index COx, total hemoglobin reactivity index THx, oxygenated hemoglobin reactivity index OHx, deoxygenated hemoglobin reactivity index DOHx, delta hemoglobin reactivity index DHx, and their ABP counterparts Mxa, COxa, THxa, OHxa, DOHxa, and DHxa.

### 2.5 Filters

Two preprocessing methods were applied to remove high‐ and low‐frequency noise from the raw signals (i.e., as acquired using a sampling frequency of 100 Hz or higher) to assess the effect on the resulting coefficients. First, we applied a low‐pass filter: elliptic filter design with a transition between 0.02 Hz and 0.2 Hz, a passband ripple allowance of 0.001 Hz, and a minimum attenuation of the stop band of 60 dB. Second, we applied a band‐pass filter by passing the output of the low‐pass filter through an additional elliptic high‐pass filter with a transition between 0.003 Hz and 0.008 Hz with the same ripple and minimum attenuation as described above. The elliptic filter design was chosen for its steep roll‐off and efficient noise separation within a narrow transition band while preserving relevant frequency content. The selected frequency band corresponds to the slow‐wave region, which is assessed through the correlation coefficients (Czosnyka et al., 2009). Zero‐phase filtering was not applied, but all signals underwent identical filtering, ensuring consistency across biosignals.

### 2.6 Power and coherence

The following additional metrics were calculated every minute over the previous 5 min of 10 s resolution data: power of slow waves for each signal (calculated as the integration of the power spectral density between the frequencies 0.005 Hz and 0.05 Hz); mean modulus coherence between ABP or CPP and either of the additional signals (e.g., ICP, CBFV, etc.) within the same frequency band. Spectral estimations were performed using the Welch method (5 segments with 50% overlap) with a Hanning data window.

### 2.7 Statistical analysis

Statistical analysis and figure preparation were performed in R Studio (R version 4.4.1—https://www.r‐project.org/—packages used: *tidyverse*, *gtsummary*, *lme4*, *cocor*, and *ggplot2*). Descriptive statistics are reported as counts/percentages, mean ± standard deviation, or median including the interquartile range as appropriate.

Considering the high variability of the different coefficients explored, to increase stability and validity of the analyses, metrics were assessed considering averages of consecutive nonoverlapping 20‐min sections. Intercorrelation was estimated using Pearson correlation. To assess the importance of ABP or CPP slow wave power (i.e., the waves which CAR aims to counteract) or of coherence on the different intercorrelations (representing the extent to which the slow‐wave power can explain the power within the brain biosignal), sections were divided into subgroups consisting of the upper or lower third of the available ranges. To compare the different correlation coefficients, Fisher's r‐to‐z transformation was applied (to acquire normally distributed z‐scores) and the resulting coefficients were then assessed using Fisher's method for comparing independent correlations for normalized sample sizes (Di Plinio, 2022; Fisher, 1921). To account for multiple comparisons, the Benjamini–Hochberg procedure (Benjamini & Hochberg, 1995) was applied limiting the proportion of false positives. The variability of the correlation coefficients depending on ABP power and coherence stratification was assessed using boxplots. Wilcoxon rank‐sum tests were used for statistical testing and correction via the Benjamini–Hochberg procedure was applied to account for multiple testing. Lastly, mixed‐effects models (Yu et al., 2022) were applied to assess the association between average values of the physiological variables (ABP, CPP, ICP, rSO_2_, and CBFV) and the coherence or ABP slow‐wave power. The patient ID was added as a random effect to adjust for patient‐specific offsets in either metric.

## 3 RESULTS

### 3.1 Patient characteristics

A total of 38 patients covering 566 h of multimodal monitoring data acquired within 97 sessions were assessed. Clinical descriptors are shown in Table 2. Median overall monitoring values were 83 (78, 90) mmHg for ABP, 12.7 (8.7, 16.3) mmHg for ICP, 72 (67, 77) mmHg for CPP, 68 (62, 72) % and 70 (66, 75) % for rSO_2_ on the left and right hemisphere, respectively, and 63 (48, 81) cm/s and 62 (43, 83) cm/s for CBFV on the left and right hemisphere, respectively.

**TABLE 2:** Patient characteristics.

	*N* = 38
Sex (male)	32 (84%)
Age	45 (35, 55)
GCS total	8 (6, 12)
Pupillary reactivity	
Both reactive	28 (74%)
One reactive	5 (13%)
None reactive	5 (13%)
Isolated TBI	9 (24%)
Decompressive craniectomy	14 (37%)
Glasgow Outcome Scale	
Good recovery	7 (18%)
Moderate disability	11 (29%)
Severe disability	10 (26%)
Dead	9 (24%)

Abbreviations: GCS, Glasgow Coma Score;TBI, traumatic brain injury.

### 3.2 Overall intercorrelation

When considering the different metrics described in Table 1, PRx displayed the best correlation to the different metrics derived from CBFV (Pearson correlation coefficient 0.42–0.47) followed by metrics derived from O_2_HB (Pearson correlation coefficient 0.32–0.34), and metrics derived from CHB and DHB (Pearson correlation coefficient 0.27–0.32). PRx displayed only a weak or no association with metrics derived from rSO_2_ (Pearson correlation coefficient 0.09–0.17) and DHB (0.01–0.08). Conversely, Mx and Mxa showed considerably weaker correlation to NIRS derived metrics, with Pearson correlation coefficients below 0.25 for metrics derived from rSO_2_, CHB, HHB, and O_2_HB.

### 3.3 Filters

**FIGURE 1:**
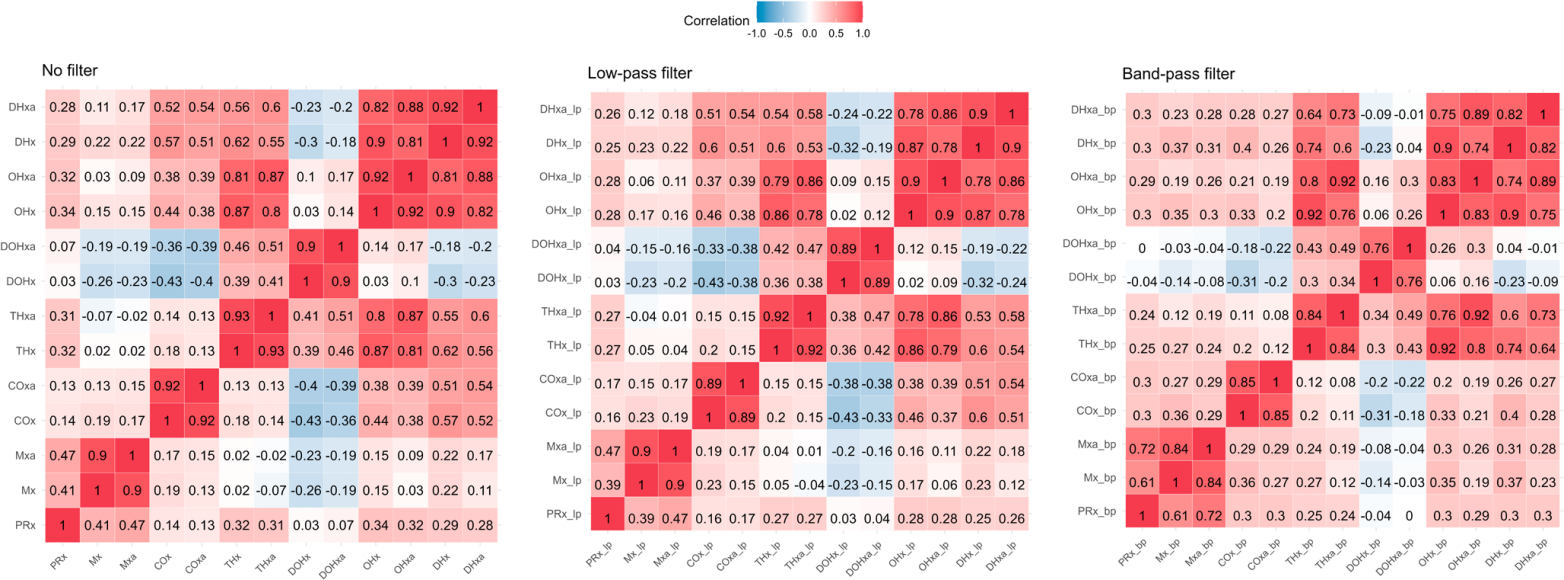
Intercorrelation depending on filter. Correlation plots are shown using the raw data (left panel), low‐pass filtered data (middle panel), and band‐pass filtered data (right panel). The color bar ranges from blue, indicating a strong negative correlation, to red, indicating a strong positive correlation. ABP, arterial blood pressure; bp, band‐pass filter; COx, cerebral oximetry index; DHx, delta hemoglobin index; DOHx, deoxygenated hemoglobin index; lp, low‐pass filter; Mx, mean flow index; OHx, oxygenated hemoglobin reactivity index; PRx, pressure reactivity index; THx, total hemoglobin index.

To evaluate the effect of filtering on the resulting metrics, correlation charts considering metrics derived from either nonfiltered, low‐pass filtered, or band‐pass filtered data were built (Figure 1). Low‐pass filtering did not alter the intercorrelations with either similar or slightly lower/higher correlation coefficients when compared to PRx. Band‐pass filtering led only to minor changes when compared to PRx.

### 3.4 Slow‐wave power

**FIGURE 2:**
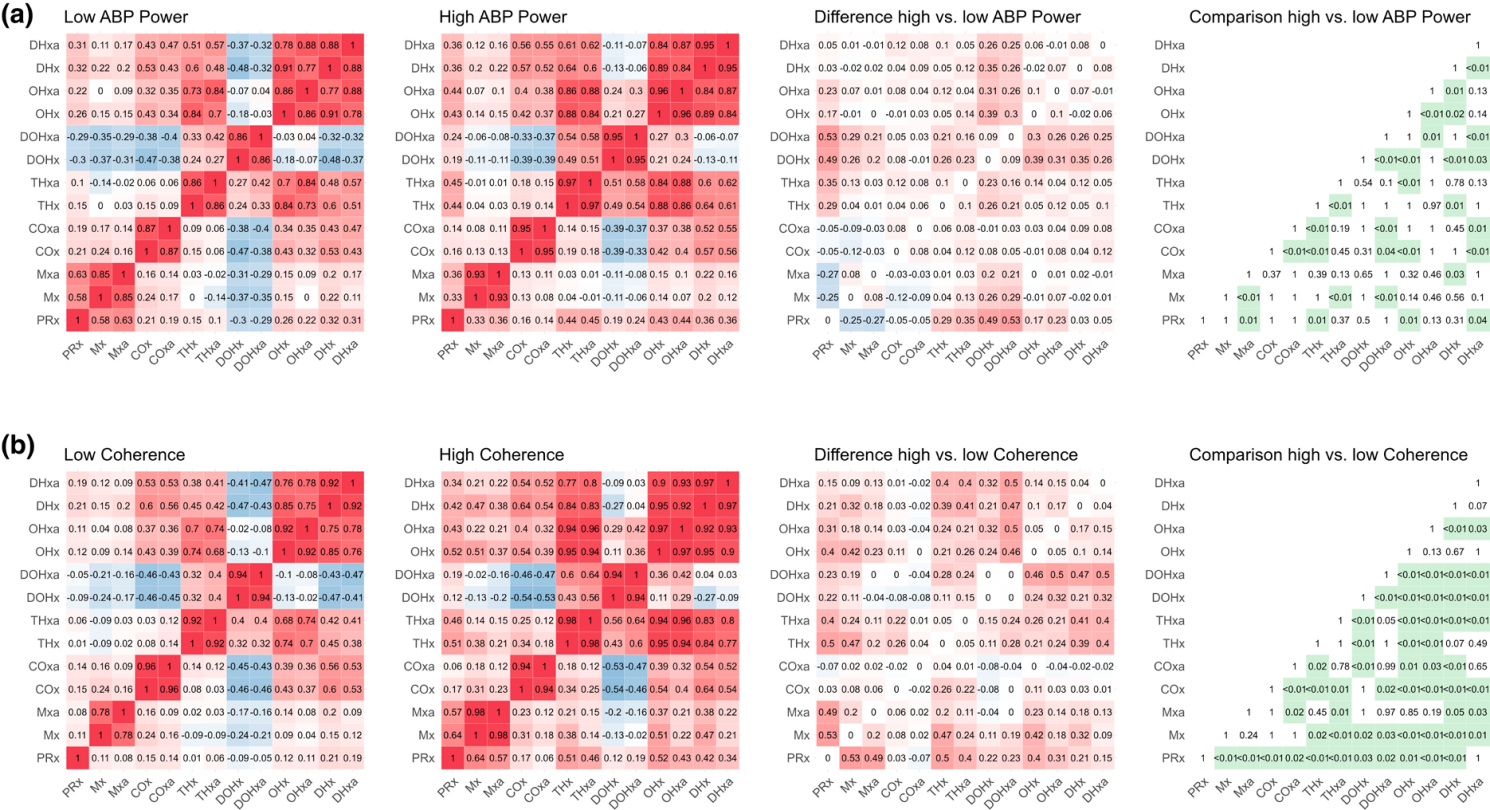
Intercorrelation depending on ABP power or coherence*. Correlation plots are shown stratified by ABP power (panel a: First panel—low ABP power; second panel—high ABP power; third panel—difference between high and low ABP power; fourth panel—comparison intercorrelation with high vs. low ABP power) or by coherence (panel b: First panel—low coherence; second panel—high coherence; third panel—difference between high and low coherence; fourth panel—comparison intercorrelation with high vs. low coherence). The color code ranges from blue, indicating a strong negative correlation, to red, indicating a strong positive correlation for the first and second panels. For the third panels, they indicate the direction of difference with red indicating an increase in intercorrelation with higher ABP power or coherence and blue a decrease in intercorrelation with higher ABP power or coherence. The fourth panels display the corresponding *p* values when comparing the intercorrelations with high and low ABP power or high and low coherence respectively. Cells with significant *p* values were shaded green. ABP, arterial blood pressure; COx, cerebral oximetry index; DHx, delta hemoglobin index; DOHx, deoxygenated hemoglobin index; Mx, mean flow index; OHx, oxygenated hemoglobin reactivity index; PRx, pressure reactivity index; THx, total hemoglobin index.

To evaluate the effect of sufficiently high ABP power on the intercorrelation, correlation charts considering only data that concurred with either the upper or lower third of present ABP power (Figure S1) were considered (Figure 2a). Sections with high ABP power were associated in some cases with higher intercorrelation between the different metrics. Overall, the largest difference was seen when considering metrics derived from NIRS‐based modalities with an increase of 0.29 for THx (*p* = 0.01) and 0.17 for OHx (*p* = 0.01) when compared to PRx. Interestingly, high ABP power was associated with a reduction in intercorrelation between PRx and Mxa (decrease of 0.27—from 0.63 to 0.36, *p* = 0.01). Similar trends were observed when compared to Mx.

### 3.5 Coherence

To evaluate the effect of sufficiently high representation of the waves within the respective power spectra, correlation charts considering only data that concurred with either the upper or lower third of present coherences (Figure S2) were built (Figure 2b). A striking difference between sections with low versus high coherences could be found. Overall, intercorrelations between PRx and other metrics of CAR were below 0.16 except for DHx when considering sections with low coherence. Conversely, sections with high coherence were associated with high correlation coefficients (Pearson correlation coefficient: 0.64 for Mx, 0.51 for THx, 0.52 for OHx, and 0.42 for DHx, *p* values of <0.01, <0.01, 0.01, <0.01, respectively). In spite of statistical significance, no meaningful difference was found when considering the rSO_2_‐derived metric COx (0.15 vs. 0.17 for low vs. high coherence respectively). Along the same lines, correlations were weak when compared to Mx within low coherence sections and considerably higher when considering high coherence sections.

### 3.6 Effect of ABP power or coherence on the absolute level of the metrics

**FIGURE 3:**
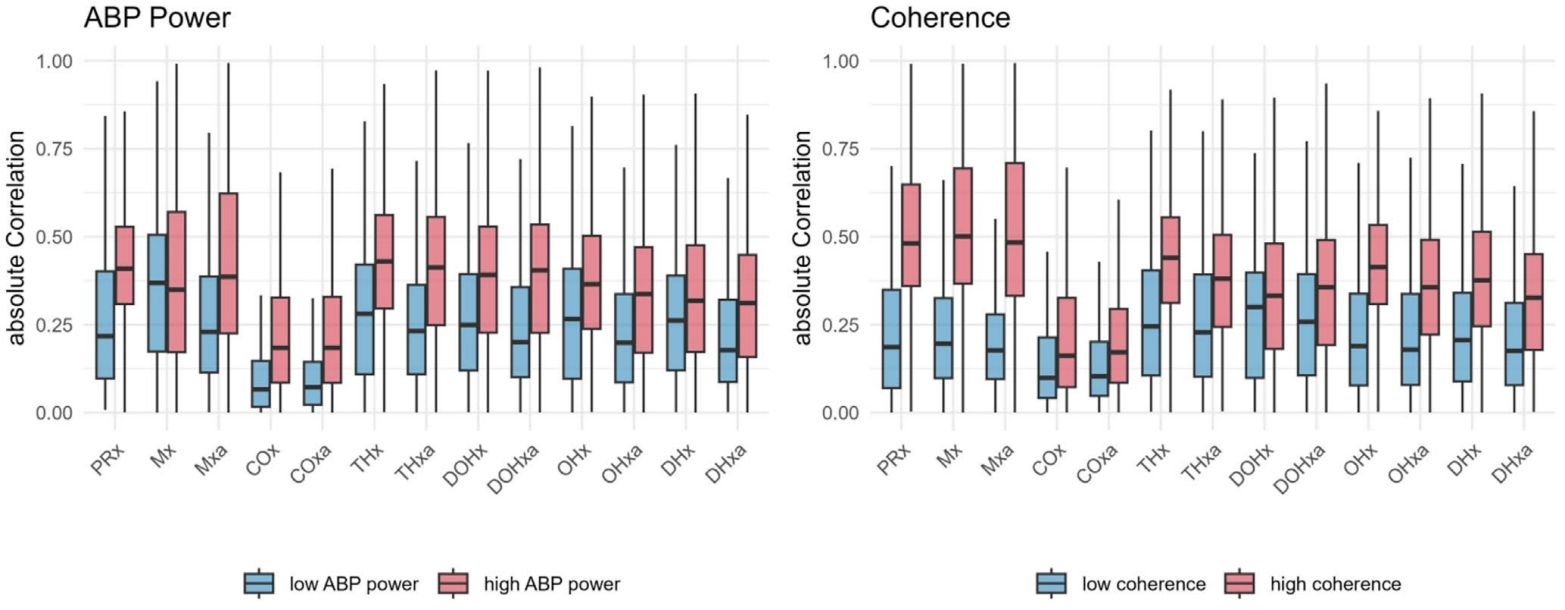
Absolute values of metrics dependent on ABP power or Coherence.* The differences in absolute correlation coefficients stratified by ABP power and coherence are illustrated using boxplots, which display the median (line inside the box), the interquartile range (upper and lower ends of the box representing the third and first quartiles, respectively), and the whiskers, which extend from the box to the largest and smallest values within 1.5 times the interquartile range from the hinges. Statistical comparison via Wilcoxon rank‐sum tests revealed significant differences (corrected using the Benjamini–Hochberg procedure) based on ABP power for all coefficients (*p* < 0.001), except for Mx, which did not significantly differ between ABP power levels. Similarly, for the stratification by Coherence, the resulting *p* values (corrected using the Benjamini–Hochberg procedure) were <0.001 in all cases. ABP, arterial blood pressure; COx, cerebral oximetry index; DHx, delta hemoglobin index; DOHx, deoxygenated hemoglobin index; Mx, mean flow index; OHx, oxygenated hemoglobin reactivity index; PRx, pressure reactivity index; THx, total hemoglobin index.

Both ABP slow‐wave power and signals' coherence were associated with distinct differences in the resulting metrics (Figure 3). Overall, high ABP power was associated with higher absolute correlation coefficients (*p* < 0.001) except for Mx, which showed no difference depending on ABP power (*p* > 0.9). Similarly, but more distinctly, high coherence between signals was associated with high correlation coefficients, mostly above 0.3 (*p* < 0.001). One exception to this pattern was COx and COxa, which overall displayed the lowest correlation coefficients even when considering sections with high ABP power or coherence.

### 3.7 Origin of ABP power and coherence differences

**FIGURE 4:**
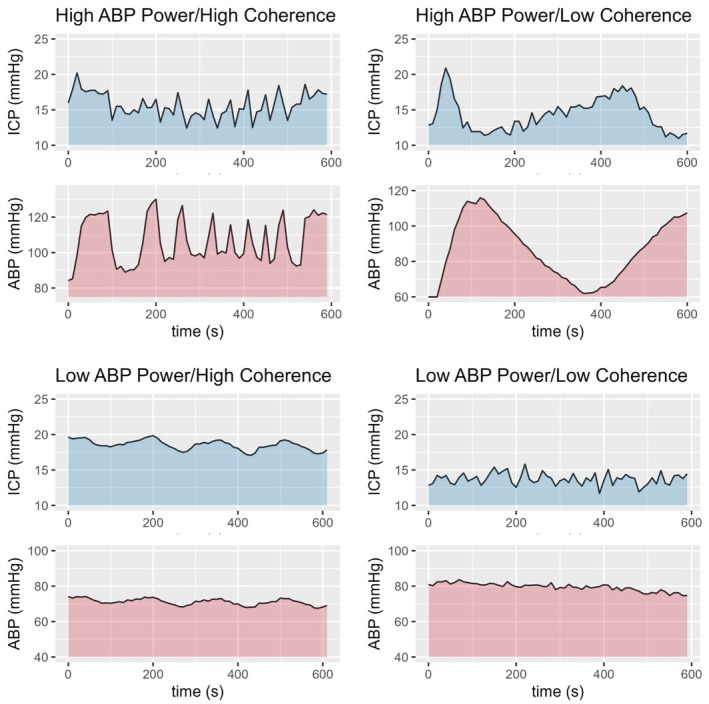
Types of concordance between ABP power and coherence. The figure displays example sections of the different combinations of high/low ABP power and coherence. It is important to note that 10‐min data sections (i.e., 600 s) are shown for better visualization. The respective metrics are derived from 300 s chunks. The perceived high coherence in the top right example is derived from changes outside of the slow‐wave range. ABP, arterial blood pressure; ICP, intracranial pressure.

Mixed‐effects models including the patient as a random effect and average values of the physiological parameters as fixed effects were applied to assess physiological predictors of high versus low ABP power or coherence (Table 3). The overarching main predictor of high ABP power or coherence was high CBFV. Additionally, high ABP was associated with high coherence between ABP and CBFV and rSO_2_. Similarly, high ICP or CPP was associated with low coherence between ABP and CBFV and rSO_2_. It is important to note that high ABP power and high coherence do not always coincide (Figure 4). The frequency with which sections with high ABP power were associated with high coherence considering the consecutive nonoverlapping 20‐min sections is shown in Table 4.

**TABLE 3:** Mixed effects models.

	Predictors			
	ABP	ICP	CPP	CBFV
ABP power	ns	ns	ns	** ↑
CBFV CPP coherence	ns	ns	ns	*** ↑
rSO_2_ CPP coherence	ns	ns	ns	ns
CHB CPP coherence	ns	ns	ns	*** ↑
O_2_HB CPP coherence	ns	ns	ns	*** ↑
HHB CPP coherence	ns	ns	ns	*** ↑
DHB CPP coherence	ns	ns	ns	*** ↑
ICP ABP coherence	ns	ns	ns	* ↑
CBFV ABP coherence	* ↑	* ↓	* ↓	*** ↑
rSO_2_ ABP coherence	* ↑	* ↓	* ↓	ns
CHB ABP coherence	ns	ns	ns	*** ↑
O_2_HB ABP coherence	ns	ns	ns	*** ↑
HHB ABP coherence	ns	ns	ns	ns
DHB ABP coherence	ns	ns	ns	*** ↑

*Note*: Mixed effects models were used to assess the association between physiological variables (ABP, CPP, ICP, rSO_2_, and CBFV) on the ABP power or coherence. The patient ID was added as a random effect to adjust for patient‐specific offsets in either metric. The results are shown by significance (non‐significant ns vs. significant asterisk) and direction of association (↑, positive, ↓, negative).

Abbreviations: ABP, arterial blood pressure; CBFV, cerebral blood flow velocity; CHB, total hemoglobin; CHB, total hemoglobin; CPP, cerebral perfusion pressure; DHB, difference oxygenated deoxygenated hemoglobin; HHB, deoxygenated hemoglobin; ICP, intracranial pressure; O_2_HB, oxygenated hemoglobin.

**TABLE 4:** Frequency of overlap between sections with high ABP power and high coherence.

	High ABP power
High CBFV CPP coherence	49%
High rSO_2_ CPP coherence	53%
High CHB CPP coherence	68%
High O_2_HB CPP coherence	66%
High HHB CPP coherence	64%
High DHB CPP coherence	64%
High ICP ABP coherence	64%
High CBFV ABP coherence	57%
High rSO_2_ ABP coherence	56%
High CHB ABP coherence	66%
High O_2_HB ABP coherence	65%
High HHB ABP coherence	67%
High DHB ABP coherence	65%

Abbreviations: ABP, arterial blood pressure; CBFV, cerebral blood flow velocity; CHB, total hemoglobin; CPP, cerebral perfusion pressure; CHB, total hemoglobin; DHB, difference oxygenated deoxygenated hemoglobin; HHB, deoxygenated hemoglobin; ICP, intracranial pressure; O_2_HB, oxygenated hemoglobin.

## 4 DISCUSSION

This study explores the agreement of different proxy measures used for the description of cerebrovascular autoregulation in relationship to the source signal characteristics in a cohort of intensive care admitted TBI patients receiving combined ICP, NIRS, and TCD monitoring. The main aim was to shed more light on why and when the different indices agree or disagree. Our analysis revealed the following main findings:

1. High or low frequency filtering only led to minor increases in intercorrelation, implying that the found differences do not arise solely due to the presence of different levels of noise or other non‐CAR related slow changes captured by the different signals.
2. High ABP slow wave power (in few cases) and high coherence between signals (consistently) increased intercorrelation. Additionally, sections with high ABP slow‐wave power and high coherence were less frequently associated with correlation coefficients around 0.

The results of this study build on previous findings by Zeiler et al. who explored differences both in terms of the absolute values as well as the prognostic information (Zeiler, Donnelly, Menon, et al., 2017). In this study, we were interested in finding methods allowing for identifying sections with higher intercorrelation between the different CAR correlation coefficients. ABP slow wave power, a previously discussed important measure to assess in CAR (Mahdi et al., 2017), describes the magnitude of the incoming slow waves, which CAR tries to counteract. Intuitively, high ABP power—representing a sufficiently high trigger of CAR—should consequently be associated with higher intercorrelation between the different CAR metrics. However, our results underline that high ABP power alone is insufficient for consistently improving intercorrelation. This might not be surprising considering that slow waves in ABP could have been dampened before being translated to changes in flow, for example, due to low compliance. This interpretation is underscored by the moderate congruence between sections with high ABP power and high coherence. Coherence, on the other hand, quantifies whether the power of incoming slow waves (input) can explain the power in the brain biosignal (output) (Claassen et al., 2016). In the absence of sufficiently high coherence, a low absolute correlation coefficient cannot be interpreted unambiguously since it is unclear whether the input variability has been efficiently attenuated so that the true response is buried in other, physiological or measurement, noises, or conversely that the main source of variability of the measured output variable is simply not the assumed input.

The analysis relies on the assumption that all the measured changes are caused by CAR or its downstream effects. The change in arteriole diameter directly reflects CAR, which then causes changes in other measures (Claassen et al., 2021). The most direct effect might be the decrease in CBFV due to the increase in distal resistance, while secondary changes caused by this increase in resistance are decreases in arterial volume (and consequently CHB and ICP). Evidently, there are other dynamic processes after TBI—such as changing metabolic demand, seizures, and edema—which might affect one measure more than the other, both due to the difference in location assessed (frontal vs. frontoparietal vs. global) as well as the measure itself (CBFV vs. volume vs. changes in hemoglobin). All of the correlation coefficients used for the description of CAR are inherently noisy (Czosnyka et al., 2009), but there are moderate positive correlations between all the different proxy measures of cerebrovascular autoregulation (Budohoski et al., 2012; Highton et al., 2015; Zeiler, Donnelly, Menon, et al., 2017). However, particularly the difference between flow versus volume regulation has previously been elucidated. Briefly, high ICP values seem to increase PRx to a higher degree than Mx (Budohoski et al., 2012). Similarly, the CPP seems to affect COx/Mx differently compared to PRx (Abecasis et al., 2021; Budohoski et al., 2012). However, since all of these correlation coefficients are calculated using different biosignals which are affected by CAR (e.g., the change in flow leads to change in ICP or hemoglobin volumes), sections with higher intercorrelation (i.e., the different correlation coefficients describe a mor similar state of CAR) might be more reliable.

One important outlier across all the analyses was COx (derived from rSO_2_). COx and its arterial counterpart COxa were neither meaningfully affected by ABP power nor coherence stratification and showed overall correlation coefficient values closest to 0, with most indices being below 0.3. Different reasons can be discussed. First, the device uses an internal protocol for the calculation of rSO_2_, which is presented with a step size of 1% and likely includes smoothing, as evidenced by the overall low coherence; although previous studies have shown that rSO_2_ can reflect large induced changes in ABP (Smith et al., 2024). The different raw hemoglobin metrics, on the other hand, follow similar changes compared to CBFV and ICP, which might be owed to the data being acquired without applying a filter or a device‐specific algorithm. Lastly, to what extent these measures represent changes in ICP compared to CBFV remains understudied.

### 4.1 Strengths and limitations

This is a single‐center study, which leads to related biases potentially limiting wider generalizability. Despite the overall large amount of data available (566 h of combined monitoring allowing for both point‐by‐point comparisons as well as time domain analyses), the patient number is relatively small. It is unclear how physiological variability or intersubject differences affect the results presented. Only patients with invasive monitoring were assessed, resulting in the description of a relatively severely affected cohort. Additionally, we only included TBI patients, which in itself is a limitation since it is unclear whether the findings are valid in other diseases or in patients with other disease characteristics and how the value of the different filtering approaches would depend on disease or disease characteristics. However, TBI represents the cohort for which these correlation coefficients are used the most. The severely affected cohort is also the one that might most likely benefit from autoregulation monitoring. We abstained from assessing the association to outcome within this cohort both due to the small sample size as well as the high variability in clinical presentation and disease trajectory. Lastly, it is important to note that increased intercorrelation between signals may not solely reflect physiological coupling but could also arise from shared noise sources or systemic artifacts affecting multiple signals simultaneously. A more in‐depth understanding of the intercorrelations could be acquired using animal studies, by exploring synthetic data, simulations, or by examining the response to induced changes in ABP or cerebral blood flow—such as those provoked by alterations in end‐tidal CO_2_ or during mean arterial pressure challenges.

## 5 CONCLUSIONS

Coherence stratification increases CAR measure intercorrelation. Since the different proxy measures all describe downstream effects of CAR, higher intercorrelation might translate to increased reliability. One outlier is COx, which displays low overall coherence to either input (ABP or CPP) and no relevant increases in intercorrelation despite the different methods applied. Future analyses should aim at assessing whether such stratification influences the calculation of clinical targets such as the optimal CPP or the lower limit of autoregulation.

## AUTHOR CONTRIBUTIONS

SYB and PS contributed to the study conception and design. Data collection was performed by SYB, GC, VM, CS, and MC. SYB performed the statistical analysis. The interpretation of the results was performed by all authors. The first draft was written by SYB. All authors commented on and revised the manuscript, and all authors read and approved the final manuscript.

## FUNDING INFORMATION

Stefan Yu Bögli is supported by the Swiss National Science Foundation (SNSF grant number: 210839/225270). Ihsane Olakorede is supported by the Cambridge Trust CIS scholarship. Erta Beqiri was supported by the Medical Research Council (grant no.: MR N013433‐1) and by the Gates Cambridge Scholarship. Claudia Ann Smith is supported by the Patrick & Margaret Flanagan Skye Cambridge Trust Scholarship. Marina Sandra Cherchi is supported by the Cantabrian Health Service (Lopez Albo Post‐Residency Program PESI/1/22).

## CONFLICT OF INTEREST STATEMENT

Peter Smielewski receives part of the licensing fees for ICM+ software, licensed by Cambridge Enterprise Ltd., University of Cambridge, Cambridge. The other authors declare no financial or nonfinancial conflicts of interest.

## ETHICS STATEMENT

The data was accessed through the Brain Physics database that is approved by the local ethics committee (REC 23/YH/0085; Yorkshire & The Humber—Leeds East Research Ethics Committee).

## CONSENT TO PARTICIPATE

Informed consent was waived by the local ethics committee.

## Supporting information


Figures S1–S2.

## Data Availability

The processed data is available upon reasonable request by the corresponding author.
