# Supplementary material for: Untangling discrepancies between cerebrovascular autoregulation correlation coefficients: An exploration of filters, coherence and power
Source: Physiol Rep. 2025 Apr 17;13(8):e70332. doi: 10.14814/phy2.70332 (PMC12004267; doi:10.14814/phy2.70332)
Supplement: Supplementary file 1 — Figures S1–S2. [file PHY2-13-e70332-s001.docx]

# **SUPPLEMENT**

**
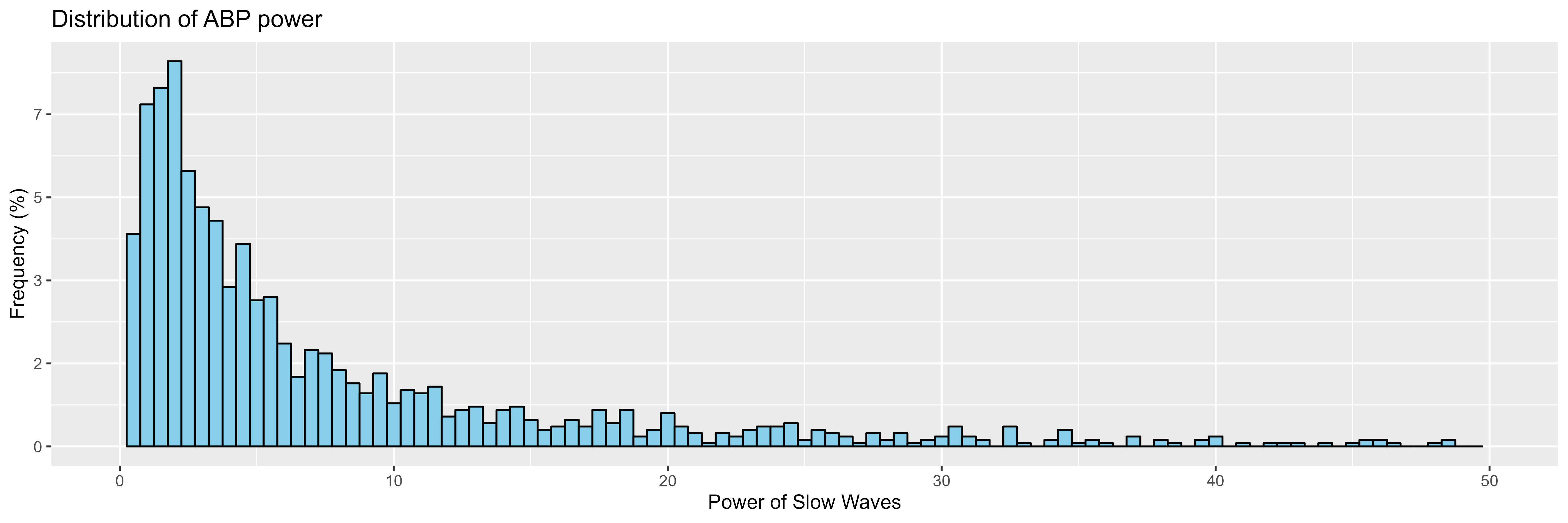
Figure S1. Arterial blood pressure slow wave power distribution.**

**Figure S2. Distribution of coherence of slow waves in measured modalities to ABP and CPP.**

**
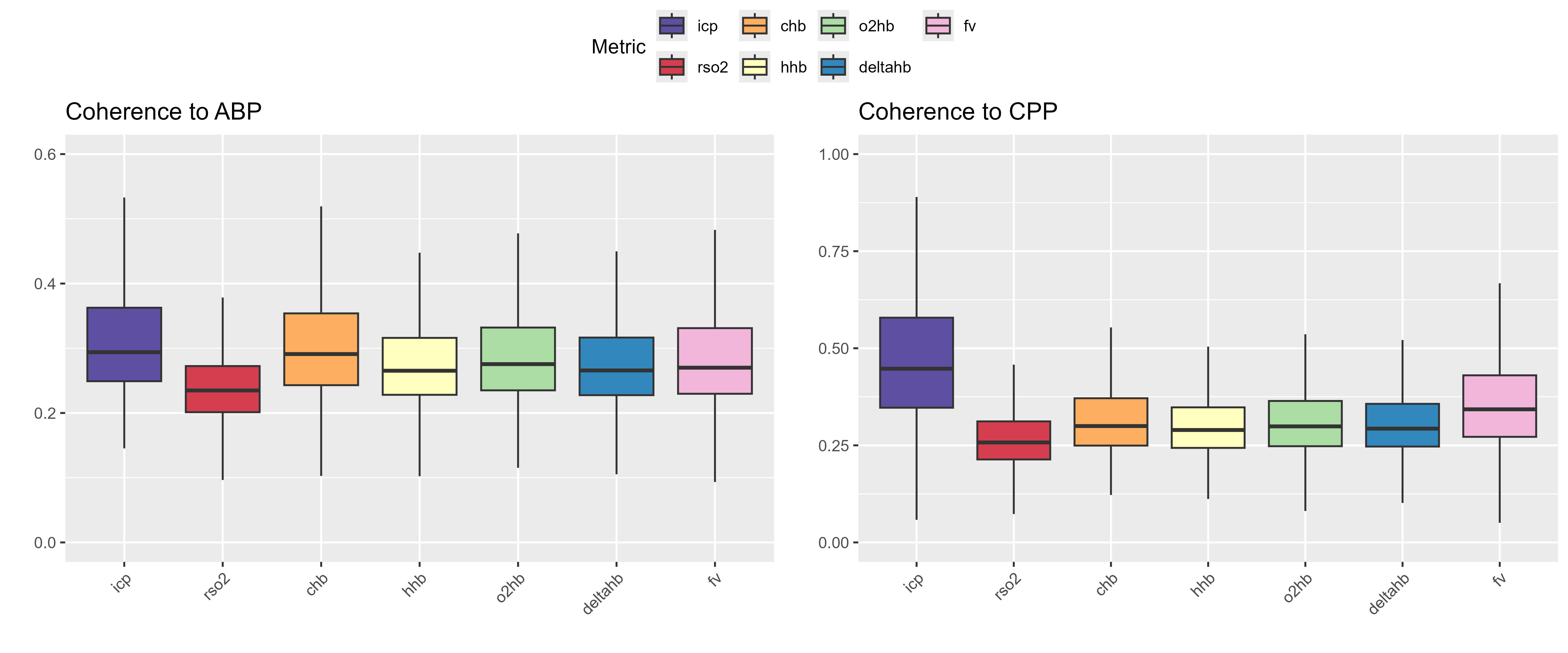
**
